# Silica Sulfuric Acid Promotes Aza-Michael Addition Reactions under Solvent-Free Condition as a Heterogeneous and Reusable Catalyst

**DOI:** 10.3390/molecules14114779

**Published:** 2009-11-23

**Authors:** Yan Wang, Yan-Qin Yuan, Sheng-Rong Guo

**Affiliations:** Department of Chemistry and Biology, Lishui University, Lishui, Zhejiang 323000, China

**Keywords:** amines, thiols, silica sulfuric acid (SSA), Michael reaction, *α,β*-unsaturated olefins

## Abstract

A highly efficient, inexpensive, recyclable, convenient, and green protocol for chemoselective aza-Michael addition reactions of amines/thiols to *α,β*-unsaturated compounds using silica sulfuric acid (SSA or SiO_2_-SO_3_H) was developed. This method is simple, convenient and the title compounds are produced in good to excellent yields.

## Introduction

The Michael reaction has been studied for over a century. The conjugate addition of amines to carbon–carbon double bonds is a useful protocol in synthetic organic chemistry [1,2,3,4]. It is used extensively in the synthesis of pharmaceutical intermediates, peptide analogues, antibiotics, and other biologically active molecules and drugs [5,6,7,8,9,10]. In the past few years, a number of alternative procedures have been developed for the conjugate addition of amines to α,β-unsaturated carbonyl compounds. In particular, various Lewis acid catalyzed reactions have been reported. This reaction has been investigated using catalysts such as lanthanum trichloride (LaCl_3_) [11], bromodimethylsulfonium bromide [12], silica supported perchloric acid [13], cerium(IV) ammonium nitrate (CAN) [14,15], β-cyclodextrin [16], zirconium(IV) chloride [17], samarium(III) triflate [18], ZrOCl_2_·8H_2_O on montmorillonite K_10_ [19], *N*-methylimidazole [20], Cu(acac)_2_ /ionic liquid [21], 1,8-diazabicyclo[5.4.0] undec-7-ene (DBU) [22], bismuth (III)triflate [23], cadmium chloride (CdCl_2_) [24], Y(NO_3_)_3_·6H_2_O [25], and cellulose supported copper(0) [26]. Unfortunately, many of these processes suffer from limitations, such as the use of expensive reagents, harsh conditions, relatively long reaction times, high catalyst loading, low selectivity and tedious work-up procedures for their separation, recycling, or disposal problems and effluent pollution. All these limitations prompted us to explore other better catalysts, which have more efficiency and less limitations. We tried to find a catalyst that has certain properties, such as the good thermal and mechanical stabilities of supported reagents, is easy to handle, of low toxicity, non-corrosive, easy to separate from reaction mixtures through filtration, and feasible for reuse.

Very recently, silica sulfuric acid (SSA) has been widely used as a reusable, heterogeneous, inexpensive solid Brønsted acid catalyst and has received much attention [27,28,29,30] and there has been increasingly awareness about the use of solid acids such as silica sulfuric acid (SSA) for synthesizing organic intermediates and fine chemicals [31,32,33,34]. SSA is a strong Brønsted and Lewis acid, presumably arising from the formation of SiO_2_-SO_3_H sites on the surface. This heterogeneous catalyst can be easily separated from the reaction media, has greater selectivity, and is recyclable, easier to handle, more stable, nontoxic, and insoluble in organic solvents. We hoped that SSA would be a superior proton source to the standard acidic solid supports for running Michael reactions under heterogeneous conditions, and herein we wish to report the use of SSA as a reusable solid acid catalyst for the Michael addition reaction.

## Results and Discussion

**Scheme 1:**
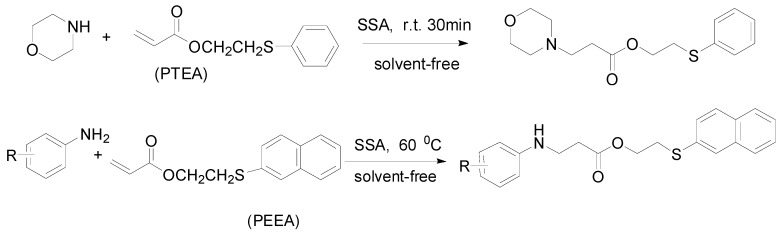
Michael addition of aliphatic and aromatic amines with PTEA or PEEA.

In a set of initial experiments, acrylic acid 2-phenylsulfanyl-ethyl ester(PTEA)was allowed to react with morpholine in the presence of a varying quantities of SiO_2_-SO_3_H. The results show that an excellent yield of the Michael adduct can be achieved by reacting a mixture of morpholine (1.2 equiv.) and PTEA (1.0 equiv) in the presence of SiO_2_-SO_3_H (SSA, 100 mg) at room temperature (Scheme 1). This encouraged us to exploit the generality and scope of this reaction catalyzed by SiO_2_-SO_3_H by using other Michael acceptors with various aromatic and aliphatic amines. As shown in Table 1, the Michael addition of various aliphatic amines and aryl amines carrying either electron-donating or electron-withdrawing groups were successfully reacted with PTEA or PEEA to produce their corresponding Michael adducts in high to excellent yields. The addition of primary amines such as benzylamine resulted in only monoalkylated products; the aromatic amines showed poor reactivity compared to the aliphatic amines. No side products were observed when using excess amines; pure products could be obtained by removal of the catalyst by filtration followed by column chromatography. The Michael addition of heteroaromatic imidazole with PTEA took more time (analysis by TLC and GC) as compared with other amines and gave a low yield of product which may be due to the weak nucleophilicity of the imidazole molecule steming from its aromaticity. The reactions with 4-nitroaniline and 2-aminophenol did not proceed at all, even at higher temperatures (entries 13, 14).

**Scheme 2:**
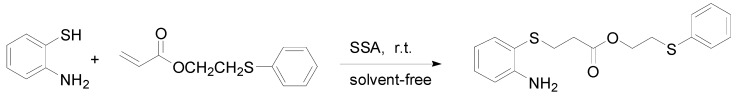
Michael addition of 2-aminobenzenethiol to PTEA.

An experiment was performed to show the chemoselectivity between thiols and aniline by using 2-aminobenzenethiol (1.2 mmol) and PTEA (1.0 mmol) in the presence of SSA (0.1 g) at room temperature. After 30 min, compound **11** was the only product (Scheme 2).

**Scheme 3:**
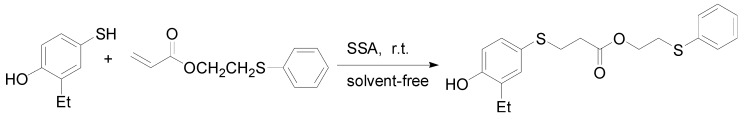
Michael addition of 3-ethyl-4-hydroxythiophenol to PTEA.

Another competition reaction was also carried out by using 3-ethyl-4- hydroxythiophenol (1.2 mmol), and PTEA (1 mmol) in the presence of SSA (0.1 g) at room temperature, and compound **12** was formed as the only product (Scheme 3). From the above two experiments, we conclude that the activity of the amines and phenols are both less than that of thiols.

We also examined the reaction of aniline with PTEA using different protic and aprotic solvents in the presence of SiO_2_-SO_3_H at different temperature. The results showed that solvent-free conditions gave a better yield of β-amino compound (Table 2).

The catalyst system was found to be recyclable and the reaction condition can be scaled up. In order to test the reusability of the catalyst, a reaction of PTEA (10 mmol) and morpholine (12 mmol) was carried out in the presence of SSA (1.0 g) and the catalyst was recovered after completion and activated by heating at 100 °C under vacuum for 1 h. The recovered catalyst was reused for the aza-Michael reaction of another batch of PTEA (10 mmol) and morpholine (12 mmol) giving 90% yield of the desired product after 1 h. Again, the catalyst was recovered, reactivated and reused repeatedly for three more consecutive times for aza-Michael reactions affording 85%, 70% and 70% yields, respectively. From this observation, it is clear that the reaction can be scaled up and the catalyst is reusable with a slight decrease in catalytic activity. 

To compare the catalytic activity of SiO_2_-SO_3_H with ordinary silica, an identical experiment was performed by stirring an intimate mixture of PTEA (1.0 mmol), morpholine (1.2 mmol) and silica (240-400 mesh, 100 mg) at room temperature. No aza-Michael addition product was formed even after 24 h, which indicate that the requirement of SiO_2_-SO_3_H is key factor for the successful outcome of the reaction. Furthermore, using of SiO_2_-SO_3_H in the aza-Michael addition reduces the cost of the catalysts, and the reaction completed in less time. It is not very clear whether SiO_2_-SO_3_H act as a solid acid catalyst or as a reservoir providing a small concentration of acid in solution. However, from the experiments carried out using recovered SiO_2_-SO_3_H suggest that it acts as a solid acid in the aza-Michael reactions.

## Experimental

### General

All reagents were purchased from commercial sources and used without further purification. TLC analysis was performed with glass backed plates precoated with silica gel and examined under UV (254 nm). NMR spectra were measured in CDCl_3_ with Me_4_Si as the internal standard on a Bruker Advance 300 instrument at room temperature. IR spectra were recorded on Bruker FT-IR spectrometer, absorbances are reported in cm^-1^. Elemental analyses were performed on a Perkin–Elmer-2400 elemental analyzer.

### General procedure for the synthesis of β-amino carbonyl compounds

A mixture of aniline (1.2 mmol) and Michael acceptor PTEA (1.0 mmol or PEEA 1.0 mmol) and SSA (0.1 g) was stirred at room temperature for 0.5–6 h. The reaction was monitored by TLC. Upon completion, EtOAc (5 mL) was added and the SiO_2_-SO_3_H catalyst can be reused after it was removed by filtration. The filtrate was washed with saturated NaHCO_3_ (aq) and brine, then dried with anhydrous Na_2_SO_4_, and concentrated under vacuum. The residue was analyzed by NMR. In most cases, pure product was obtained. If needed, the residue was further purified by silica gel column chromatography using a 4:1 hexane–EtOAc mixture as eluent to afford the pure product. All of the products are new compounds, and the structures of products were determined from their NMR, MS or elemental analyses. 

### Selected spectroscopic data

*3-Phenylaminopropionic acid 2-phenylsulfanyl-ethyl ester* (**1**): yellow oil; ^1^H-NMR *δ*: 7.42 (d, *J* = 7.7 Hz, 2H), 7.18-7.34 (m, 5H), 6.75 (t, *J* = 7.3 Hz, 1H), 6.62 (d, *J* = 8.0 Hz, 2H), 4.30 (t, *J* = 6.8 Hz, 2H), 3.95 (br 1H), 3.46 (t, *J* = 6.3 Hz, 2H), 3.17 (t, *J* = 6.8 Hz, 2H), 2.62 (t, *J* = 6.3 Hz, 2H); ^13^C-NMR *δ*: 172.1, 147.5, 134.9, 130.1, 129.3, 129.1, 126.7, 117.8, 113.1, 63.0, 39.4, 33.9, 32.5; Anal. Calcd. for C_17_H_19_NO_2_S: C, 67.74; H, 6.35. Found: C, 67.76; H, 6.34.

*3-p-Tolylaminopropionic acid 2-phenylsulfanyl-ethyl ester* (**2**): yellow oil; ^1^H-NMR *δ*: 7.43-7.21 (m, 5H), 7.02 (d, *J* = 8.2 Hz, 2H), 6.58 (d, *J* = 8.2Hz, 2H), 4.29 (t, *J* = 6.8 Hz, 2H), 3.93 (br, 1H), 3.44 (t, *J* = 6.3 Hz, 2H), 3.16 (t, *J* = 6.8 Hz, 2H), 2.60 (t, *J* = 6.2 Hz, 2H), 2.27(s, 3H); ^13^C-NMR δ: 172.2, 145.2, 134.9, 130.1, 129.8, 129.1, 127.0, 126.7, 113.3, 62.9, 39.8, 33.8, 32.5, 20.4; Anal. Calcd. for C_18_H_21_NO_2_S: C, 68.54; H, 6.71. Found: C, 68.56; H, 6.68.

*3-(4-Methoxyphenylamino)-propionic acid 2-phenylsulfanyl-ethyl ester* (**3**): Off-white oil; ^1^H-NMR *δ*: 7.23-7.42 (m, 5H), 6.80 (q, *J* = 2.3 Hz, *J* = 6.9 Hz, 2H), 6.62 (d, *J* = 2.3 Hz, *J* = 6.9 Hz, 2H), 4.29 (t, *J* = 6.8 Hz, 2H), 3.76 (s, 3H), 3.43 (br, 1H), 3.40 (t, *J* = 6.3 Hz, 2H), 3.16 (t, *J* = 6.8 Hz, 2H), 2.58 (t, *J* = 6.3 Hz, 2H); ^13^C-NMR *δ*: 172.2, 152.5, 141.7, 135.0, 129.8, 129.0, 126.7, 114.9, 114.6, 63.9, 55.8, 40.5, 33.9, 32.5; Anal. Calcd. for C_18_H_21_NO_3_S: C, 65.23; H, 6.39. Found: C, 65.15; H, 6.34.

*3-(4-Chlorophenylamino)propionic acid 2-phenylsulfanyl-ethyl ester* (**4**): Off-white oil; ^1^H-NMR *δ*: 7.41 (d, *J* = 1.5 Hz, 2H), 7.23-7.39 (m, *J* = 6.4 Hz, 3H), 7.13 (t, *J* = 2.2 Hz, 2H), 6.59 (t, *J* = 2.0 Hz, 2H), 4.29 (t, *J* = 6.8 Hz, 2H), 3.97 (br, 1H), 3.42 (t, *J* = 6.2 Hz, 2H), 3.16 (t, *J* = 6.8 Hz, 2H), 2.58 (t, *J* = 6.2, 2H); ^13^C-NMR *δ*: 171.0, 143.9, 136.4, 129.6, 129.3, 128.9, 126.1, 125.1, 114.9, 63.6, 39.0, 37.8, 35.7; Anal. Calcd. for C_17_H_18_ClNO_2_S: C, 60.80; H, 5.40. Found: C, 60.83; H, 5.42.

*3-(3,4-Dichlorophenylamino)propionic acid 2-phenylsulfanyl-ethyl ester* (**5**): yellow oil; ^1^H-NMR *δ*: 7.56-7.78 (m, 3H), 7.14-7.21 (m, 3H), 6.71 (d, *J* = 8.2 Hz, 2H), 5.59 (br, 1H), 4.27 (t, *J* = 6.8 Hz, 2H), 3.36 (t, *J* = 6.3 Hz, 2H), 3.12 (t, *J* = 6.8 Hz, 2H), 2.60 (t, *J* = 6.3 Hz, 2H); ^13^C-NMR *δ*: 172.3, 155.8, 136.1, 134.5, 132.9, 131.4, 131.1, 130.6, 127.6, 124.2, 116.4, 63.2, 34.5 , 31.4, 22.5; Anal. Calcd. for C_17_H_17_Cl_2_NO_2_S: C, 55.14; H, 4.63. Found: C, 55.10; H, 4.65.

*3-Morpholin-4-ylpropionic acid 2-phenylsulfanyl-ethyl ester* (**6**): Off-white oil; ^1^H-NMR *δ*: 7.42-7.38 (m, 2H), 7.28-7.34 (m, 2H), 7.25-7.22 (m, 1H), 4.27 (t, *J* = 6.8 Hz, 2H), 3.73-3.71 (m, 4H), 3.15 (t, *J* = 6.8 Hz, 2H), 2.71 (t, *J* = 7.2 Hz, 2H), 2.38-2.46 (m, 6H); ^13^C-NMR *δ*: 171.9, 135.1, 129.9, 129.1, 126.6, 66.5, 63.0, 53.6, 53.1, 32.4, 31.4; Anal. Calcd. for C_15_H_21_NO_3_S: C, 60.99; H, 7.17. Found: C, 60.97; H, 7.16.

*3-Diethylamino-propionic acid 2-phenylsulfanyl-ethyl ester* (**7**): Off-white oil; ^1^H-NMR *δ*: 7.13-7.28 (m, 5H), 4.46 (t, *J* = 6.8 Hz, 2H), 3.74 (t, *J* = 6.3 Hz, 2H), 3.12 (t, *J* = 6.8 Hz, 2H), 2.78 (t, *J* = 6.3 Hz, 2H), 2.43 (q, *J* = 6.2 Hz, 4H), 1.10 (t, *J* = 6.2 Hz, 6H); ^13^C-NMR *δ*: 172.1, 135.6, 128.7, 126.6, 124.9, 66.5, 48.6, 46.8, 53.1, 34.8, 16.7; Anal. Calcd. for C_15_H_23_NO_2_S: C, 64.02; H, 8.24. Found: C, 64.04; H, 8.25.

*3-Piperidin-1-yl-propionic acid 2-phenylsulfanyl-ethyl ester* (**8**): Off-white oil; ^1^H-NMR *δ*: 7.75-7.85 (m, 3H), 7.43-7.51 (m, 2H), 4.33 (t, *J* = 6.8 Hz, 2H), 3.68 (t, *J* = 7.2 Hz, 2H), 2.63 (t, *J* = 6.8 Hz, 2H), 2.44 (t, *J* = 7.2 Hz, 2H), 2.34 (br, 10H); ^13^C-NMR *δ*: 171.9, 135.1, 129.9, 129.1, 126.6, 66.5, 63.0, 53.6, 53.1, 32.4, 31.4, 29.8; Anal. Calcd. for C_16_H_23_NO_2_S: C, 65.49; H, 7.90. Found: C, 65.51; H, 7.91.

*3-Benzylaminopropionic acid 2-phenylsulfanyl-ethyl ester* (**9**): Off-white oil; ^1^H-NMR *δ*: 7.21-7.38 (m, 10H), 4.27 (t, *J* = 6.8 Hz, 2H), 3.81(s, 2H), 3.16 (t, *J* = 6.3 Hz, 2H), 2.90 (t, *J* = 6.8 Hz, 2H), 2.52 (t, *J* = 6.3 Hz, 2H); ^13^C-NMR *δ*: 172.5, 140.1, 135.1, 130.0, 129.1, 128.5, 128.0, 127.0, 126.6, 62.8, 53.8, 44.4, 34.7, 32.4; Anal. Calcd. for C_18_H_21_NO_2_S: C, 68.54; H, 6.71. Found: C, 68.53; H, 6.68.

*3-Imidazol-1-yl-propionic acid 2-phenylsulfanyl-ethyl ester* (**10**): Off-white oil; ^1^H-NMR *δ*: 7.56 (s, 1H), 6.97-7.09 (m, 5H), 6.87 (d, *J* = 7.3 Hz, 2H), 4.36 (t, *J* = 6.8 Hz, 2H), 3.96 (t, *J* = 6.3 Hz, 2H), 3.16 (t, *J* = 6.8 Hz, 2H), 2.60 (t, *J* = 6.3 Hz, 2H); ^13^C-NMR *δ*: 170.2, 141.3, 129.9, 127.9., 126.7, 125.2, 124.8, 122.9, 65.6, 39.5, 32.4, 31.6; Anal. Calcd. for C_14_H_16_N_2_O_2_S: C, 60.85; H, 5.84. Found: C, 60.87; H, 5.82. 

*3-(2-Aminophenylsulfanyl)propionic acid 2-phenylsulfanyl-ethyl ester* (**11**): Off-white Oil; ^1^H-NMR *δ*: 7.15-7.42 (m, *J* = 7.3 Hz, 7H), 6.67-6.75 (m, 2H), 4.40 (br, 2H), 4.25 (t, *J* = 6.8 Hz, 2H), 3.15 (t, *J* = 6.3 Hz, 2H), 2.96 (t, *J* = 6.8 Hz, 2H), 2.54 (t, *J* = 6.3 Hz, 2H); ^13^C-NMR *δ*: 171.7, 148.7, 136.6, 135.0, 130.2, 130.0, 129.1, 126.7, 118.5, 116.4, 115.0, 63.2, 34.5, 32.4, 29.5; Anal. Calcd. for C_17_H_19_NO_2_S_2_: C, 61.23; H, 5.74. Found: C, 61.25; H, 5.72.

*3-(3-Ethyl-4-hydroxyphenylsulfanyl)propionic acid 2-phenylsulfanyl-ethyl ester* (**12**): yellow oil; ^1^H- NMR *δ*: 7.15-7.43 (m, 6H), 6.69-6.72 (m, *J* = 8.2 Hz, 2H), 5.56 (br, 1H), 4.27 (t, *J* = 6.8 Hz, 2H), 3.15 (t, *J* = 6.3 Hz, 2H), 3.02 (t, *J* = 6.8 Hz, 2H), 2.54-2.66 (m, 4H), 1.24 (t, *J* = 7.6 Hz, 3H); ^13^C-NMR *δ*: 172.1, 153.6, 135.0, 134.1, 131.7, 131.2, 130.0, 129.1, 126.7, 124.7, 115.9, 63.2, 34.5, 32.4, 31.1, 22.9, 13.9; Anal. Calcd. for C_19_H_22_O_3_S_2_: C, 62.95; H, 6.12. Found: C, 62.92; H, 6.14.

*3-Phenylaminopropionic acid 2-(naphthalen-2-ylsulfanyl)-ethyl ester* (**13**): yellow oil; ^1^H-NMR *δ*: 7.73-7.81 (m, 4H), 7.42-7.52 (m, 4H), 7.04 (d, *J* = 8.1 Hz, 2H), 6.45 (d, *J* = 8.1 Hz, 2H), 4.29 (t, *J* = 6.7 Hz, 2H), 3.91 ( br, 1H), 3.41 (t, *J* = 6.3 Hz, 2H), 3.24 (t, *J* = 6.7 Hz, 2H), 2.57 (t, *J* = 6.3 Hz, 2H); ^13^C-NMR *δ*: 172.0, 145.6, 132.9, 132.6, 131.7, 129.9, 128.2, 128.2, 127.9, 127.5, 127.2, 126.4, 126.2, 116.7, 112.8, 63.4, 39.5, 33.3, 32.1; Anal. Calcd. for C_21_H_21_NO_2_S: C, 71.76; H, 6.02. Found: C, 71.79; H, 6.00.

*3-p-Tolylaminopropionic acid 2-(naphthalen-2-ylsulfanyl)-ethyl ester* (**14**): white solid, m.p. 75-77 °C; ^1^H-NMR *δ*: 7.76-7.83 (m, 4H), 7.49 (t, *J* = 8.4 Hz, 3H), 7.01 (d, *J* = 8.1 Hz, 2H), 6.56 (d, *J* = 8.1 Hz, 2H), 4.32 (q, *J* = 6.7 Hz, 2H), 3.90 ( br, 1H), 3.41 (t, *J* = 6.3 Hz, 2H), 3.25 (m, *J* = 6.7 Hz, 2H), 2.57 (t, *J* = 6.3 Hz, 2H), 2.51 (s, 3H); ^13^C-NMR *δ*: 172.2, 145.2, 133.7, 132.4, 132.0, 129.8, 128.7, 128.2, 128.1, 127.7, 127.2, 127.0, 126.7, 126.0, 113.4, 62.9, 39.8, 33.9, 32.4, 20.4; Anal. Calcd. for C_22_H_23_NO_2_S: C, 72.30; H, 6.34. Found: C, 72.32; H, 6.36.

*3-(4-Methoxyphenylamino)propionic acid 2-(naphthalen-2-ylsulfanyl)-ethyl ester* (**15**): Off-white oil; ^1^H-NMR *δ*: 7.78-7.89 (m, 4H), 7.46-7.54 (m, 3H), 6.82 (d, *J* = 8.8 Hz, 2H), 6.63 (d, *J* = 8.8 Hz, 2H), 4.35 (t, *J* = 6.8 Hz, 2H), 3.82 (br, 1H), 3.75 (s, 3H), 3.38 (t, *J* = 6.3 Hz, 2H), 3.27 (t, *J* = 6.3 Hz, 2H), 2.58 (t, *J* = 6.8 Hz, 2H); ^13^C-NMR *δ*: 172.2, 152.6, 141.7, 133.8, 132.5, 132.0, 128.7, 128.0, 127.9, 127.7, 127.2, 126.7, 126.0, 115.1, 114.8, 63.1, 55.8, 40.6, 33.9, 32.3; Anal. Calcd. for C_22_H_23_NO_3_S: C, 69.26; H, 6.08. Found: C, 69.29; H, 6.05.

*3-Morpholin-4-yl-propionic acid 2-(naphthalen-2-ylsulfanyl)-ethyl ester* (**16**): yellow oil; ^1^H-NMR *δ*: 7.73-7.83 (m, 4H), 7.40-7.49 (m, 3H), 4.31 (t, *J* = 6.8 Hz, 2H), 3.67 (t, *J* = 4.6 Hz, 4H), 3.23 (t, *J* = 6.8 Hz, 2H), 2.64 (t, *J* = 7.3 Hz, 2H), 2.41-2.46 (tt, *J* = 7.3 Hz, *J* = 4.6 Hz, 6H); ^13^C-NMR *δ*: 171.0, 135.1, 133.9, 128.3, 127.2, 126.5, 126.3, 126.1, 124.7, 66.5, 63.0, 53.6, 53.1, 32.4, 31.4; Anal. Calcd. for C_19_H_23_NO_3_S: C, 66.06; H, 6.71. Found: C, 66.08; H, 6.73.

## Conclusions

In conclusion, we have described herein the scope and limitations of the use of silica sulfuric acid (SSA or SiO_2_-SO_3_H) as a highly efficient catalyst for Michael additions of amines under solvent-free conditions. This process avoids the use of organic solvent to carry out the reaction, and the catalyst can be separated from the product very easily after the reaction. Handling of the catalyst is easy, and it can be used again.

## Figures and Tables

**Table 1:** Michael addition of amines catalyzed by SSA under solvent-free conditions.

Entry	Amine	Michael acceptor	Product	Time (h)	Yield (%) *^b^*
1	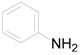	PTEA	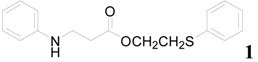	0.5	92
2	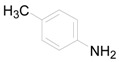	PTEA	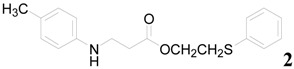	1.5	88
3	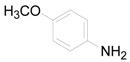	PTEA	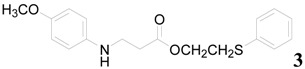	0.5	90
4	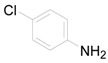	PTEA	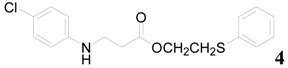	1.0	96
5	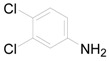	PTEA	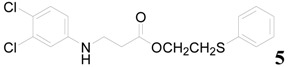	1.5	85
6	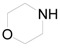	PTEA	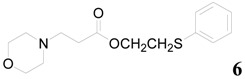	0.5	95
7	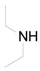	PTEA	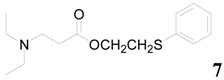	1.5	93
8	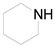	PTEA	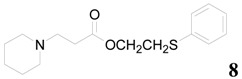	1.5	88
9	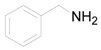	PTEA	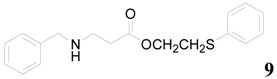	2.0	86
10	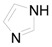	PTEA	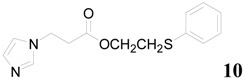	4.0	12
11	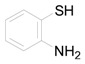	PTEA	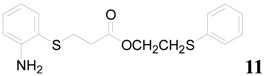	0.5	94
12	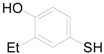	PTEA	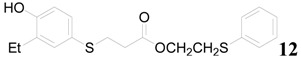	0.5	91
13	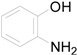	PTEA	No Reaction		
14	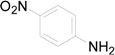	PTEA	No Reaction		
15	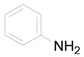	PEEA	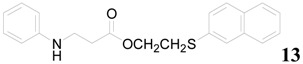	2.5	86
16	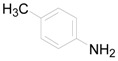	PEEA	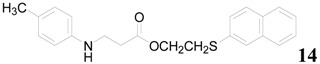	3.0	90
17	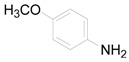	PEEA	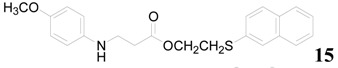	1.0	89
18	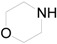	PEEA	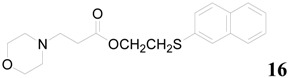	0.5	92

**Table 2:** Michael addition reaction of PTEA with aniline in protic and aprotic solvents.

Entry	Solvent	Yield (%)
1 2 3 4 5 6 7 8	C_2_H_5_OH H_2_O CH_3_CO_2_H ClCH_2_CH_2_Cl Toluene CH_3_CN CH_2_Cl _2_ Neat	72 34 78 83 41 86 86 91
